# Molecular Detection of *Cryptosporidium* spp. and *Enterocytozoon bieneusi* Infection in Wild Rodents From Six Provinces in China

**DOI:** 10.3389/fcimb.2021.783508

**Published:** 2021-11-25

**Authors:** Hong-Bo Ni, Yu-Zhe Sun, Si-Yuan Qin, Yan-Chun Wang, Quan Zhao, Zheng-Yao Sun, Miao Zhang, Ding Yang, Zhi-Hui Feng, Zheng-Hao Guan, Hong-Yu Qiu, Hao-Xian Wang, Nian-Yu Xue, He-Ting Sun

**Affiliations:** ^1^ College of Life Science, Changchun Sci-Tech University, Shuangyang, China; ^2^ College of Veterinary Medicine, Qingdao Agricultural University, Qingdao, China; ^3^ State Key Laboratory of Veterinary Etiological Biology, Key Laboratory of Veterinary Parasitology of Gansu Province, Lanzhou Veterinary Research Institute, Chinese Academy of Agricultural Sciences, Lanzhou, China; ^4^ Center of Prevention and Control Biological Disaster, State Forestry and Grassland Administration, Shenyang, China; ^5^ Veterinary Department, Muyuan Foods Co., Ltd., Nanyang, China; ^6^ College of Animal Science and Veterinary Medicine, Heilongjiang Bayi Agricultural University, Daqing, China

**Keywords:** *Cryptosporidium* spp., *Enterocytozoon bieneusi*, prevalence, genotyping, wild rats, China

## Abstract

*Enterocytozoon* (*E.*) *bieneusi* and *Cryptosporidium* spp. are the most important zoonotic enteric pathogens associated with diarrheal diseases in animals and humans. However, it is still not known whether *E. bieneusi* and *Cryptosporidium* spp. are carried by wild rodents in Shanxi, Guangxi, Zhejiang, Shandong, and Inner Mongolia, China. In the present study, a total of 536 feces samples were collected from *Rattus (R.) norvegicus*, *Mus musculus*, *Spermophilus* (*S.*) *dauricus*, and *Lasiopodomys brandti* in six provinces of China, and were detected by PCR amplification of the SSU rRNA gene of *Cryptosporidium* spp. and ITS gene of *E. bieneusi* from June 2017 to November 2020. Among 536 wild rodents, 62 (11.6%) and 18 (3.4%) samples were detected as *E. bieneusi*- and *Cryptosporidium* spp.-positive, respectively. Differential prevalence rates of *E. bieneusi* and *Cryptosporidium* spp. were found in different regions. *E. bieneusi* was more prevalent in *R. norvegicus*, whereas *Cryptosporidium* spp. was more frequently identified in *S. dauricus*. Sequence analysis indicated that three known *Cryptosporidium* species/genotypes (*Cryptosporidium viatorum*, *Cryptosporidium felis*, and *Cryptosporidium* sp. rat genotype II/III) and two uncertain *Cryptosporidium* species (*Cryptosporidium* sp. novel1 and *Cryptosporidium* sp. novel2) were present in the investigated wild rodents. Meanwhile, 5 known *E. bieneusi* genotypes (XJP-II, EbpC, EbpA, D, and NCF7) and 11 novel *E. bieneusi* genotypes (ZJR1 to ZJR7, GXM1, HLJC1, HLJC2, and SDR1) were also observed. This is the first report for existence of *E. bieneusi* and *Cryptosporidium* spp. in wild rodents in Shanxi, Guangxi, Zhejiang, and Shandong, China. The present study also demonstrated the existence of *E. bieneusi* and *Cryptosporidium* spp. in *S. dauricus* worldwide for the first time. This study not only provided the basic data for the distribution of *E. bieneusi* and *Cryptosporidium* genotypes/species, but also expanded the host range of the two parasites. Moreover, the zoonotic *E. bieneusi* and *Cryptosporidium* species/genotypes were identified in the present study, suggesting wild rodents are a potential source of human infections.

## Introduction

The rodents are one of the largest families of mammals. Wild rodents (e.g., wild rats) are the most widely distributed worldwide. They can shed many pathogens (e.g., *Enterocytozoon* (*E.*) *bieneusi* and *Cryptosporidium* spp.) into the environment due to living in an open environment, thus becoming potential sources for transmission of pathogens to other animals (Deng et al., 2016; García-Livia et al., 2020; Gui et al., 2020). In addition, the rodents have a closed relationship with humans. Thus, many pathogens, including *E. bieneusi* and *Cryptosporidium* spp., might be transmitted from rodents to humans. (García-Livia et al., 2020; Gui et al., 2020; Zhao et al., 2020).

*E. bieneusi* and *Cryptosporidium* spp. are the common zoonotic enteric pathogens responsible for a majority of parasitic diarrhea diseases worldwide (Qi et al., 2015; Zhang X. et al., 2018; Zhao et al., 2018; Wang S. N. et al., 2020). Both of them can infect humans and a wide variety of animals (e.g., rodents) (Wang et al., 2013; Zhao et al., 2018; Li and Xiao, 2020; Wang S. N. et al., 2020) mainly through water-borne and food-borne routes (Wang et al., 2013; Zhao et al., 2018). In general, healthy people infected with both pathogens are asymptomatic or manifest symptoms of self-limiting diarrhea. However, the infection of *E. bieneusi* and *Cryptosporidium* spp. in immunocompromised individuals may cause chronic or life-threatening diarrheas (Wang et al., 2013; Sutthikornchai et al., 2021). Owing to their significance in public health, *Cryptosporidium* spp. and *E. bieneusi* have been put into Category B Priority Pathogen list by the National Institute of Allergy and Infectious Diseases (NIAID) (NIAID, 2018). Moreover, *E. bieneusi* is also listed on the Environmental Protection Agency (EPA) microbial contaminant candidate list of concern for waterborne transmission (Didier et al., 2009).

*E. bieneusi* is consist of more than 500 genotypes, which are classified into 11 groups based on the sequences of the internal transcribed spacer (ITS) region of the rRNA gene (Santin, 2015;  Zhang Y. et al., 2018; Zhao et al., 2018; Li W. et al., 2019; Wang S. N. et al., 2020; Abarca et al., 2021). Group 1, identified as zoonotic, is responsible for a vast majority of human infections (Wang S. N. et al., 2020). Groups 2-11 are mainly composed of host-specific or host-adapted genotypes (Guo et al., 2014; Wang S. N. et al., 2020). To date, a total of 36 ITS genotypes of *E. bieneusi* have been found in rodent species and 15 (Type IV, BEB6, EbpA, EbpC, C, D, H, CZ3, S6, Peru6, Nig7, Peru8, Peru11, Peru16, and PigITS5) were considered as zoonotic genotypes (Danišová et al., 2015; Cama et al., 2007; Sak et al., 2011; Guo et al., 2014; Perec-Matysiak et al., 2015; Qi et al., 2015; Roellig et al., 2015; Deng et al., 2016).

*Cryptosporidium* spp. contains more than 100 species/genotypes based on the sequence of the small subunit (SSU) rRNA gene (Feng et al., 2018; Holubová et al., 2019). To date, 38 of them have been identified in humans, whereas only *C. hominis* and *C. parvum* were frequently found in humans (Essid et al., 2018; Krumkamp et al., 2021), and the remaining genotypes/species were occasionally observed in humans. Rodents are one of the most important reservoirs of *Cryptosporidium* spp. More than 30 *Cryptosporidium* species/genotypes have been identified in rodent species (Zhang X. et al., 2018). Among them, at least ten *Cryptosporidium* species (including *C. parvum*, *C. andersoni*, *C. muris*, *C. wrairi*, *C. tyzzeri*, *C. scrofarum*, *C. ubiquitum*, *C. hominis*, *C. suis*, and *C. meleagridis*) and more than 20 *Cryptosporidium* genotypes, such as ground squirrel genotypes (I-III), rat genotypes (I-IV), deer mouse genotypes (I-IV), chipmunk genotypes II, vole genotype, and mouse genotypes (II, III), have been identified in humans (Bajer et al., 2002; Nakai et al., 2004; Feng et al., 2007; Foo et al., 2007; Kimura et al., 2007; Kvác et al., 2008; Lv et al., 2009; Paparini et al., 2012; Backhans et al., 2013; Murakoshi et al., 2013; Ng-Hublin et al., 2013; Song et al., 2015; Stenger B. et al., 2015; Stenger B. L. et al., 2015; Zhao et al., 2015; Gholipoury et al., 2016; Saki et al., 2016; Danišová et al., 2017; Wang S. N. et al., 2020).

In view of such severe situations, it is essential to investigate the prevalence of *E. bieneusi* and *Cryptosporidium* spp. in different rodent species and identify their species/genotypes. However, information regarding *Cryptosporidium* spp. infection in rodents was limited in China, which was only reported in *Microtus fuscus* (Qinghai vole) and *Ochotona curzoniae* (wild plateau pika) in Qinghai (Zhang X. et al., 2018), brown rats (*Rattus norvegicus*) in Heilongjiang (Li et al., 2016), bamboo rats in Sichuan (Liu et al., 2015), pet chinchillas in Beijing, Henan and Guizhou (Qi et al., 2015), commensal rodents in Henan and Fujian (Zhao et al., 2015), brown rats in Heilongjiang (Zhao et al., 2018), wild, laboratory, and pet rodents in Beijing, Henan, Fujian and Sichuan (Lv et al., 2009), bamboo rats in Guangdong, Hunan, Guangxi, Jiangxi and Hainan (Wei et al., 2019; Li et al., 2020a; Li et al., 2020b), Asian house rats, brown rats, Edward’s long-tailed rats and muridae in Hainan (Zhao et al., 2019). In China, *E. bieneusi* in rodents has been only reported in Heilongjiang (Zhao et al., 2018), Beijing (Qi et al., 2015), Henan (Qi et al., 2015; Wang J. et al., 2020), Guizhou (Qi et al., 2015), Sichuan (Deng et al., 2016), Shandong (Wang J. et al., 2020), Guangdong (Wang et al., 2019), Hunan (Wang et al., 2019; Gui et al., 2020), Jiangxi (Wang et al., 2019), Chongqing (Wang et al., 2019), Guangxi (Wang et al., 2019), and Hainan (Zhao et al., 2020).

However, it is still not known whether *E. bieneusi* and *Cryptosporidium* spp. are carried by wild rodents in Shanxi, Guangxi, Zhejiang, Shandong, and Inner Mongolia, China. Thus, the present study was performed to estimate the prevalence and genotypes of *E. bieneusi* and *Cryptosporidium* spp. in wild rodents by the molecular biological method.

## Materials and Methods

### Specimen Collection

A total of 536 feces samples were collected from four rodent species from Daqing City in Heilongjiang (n = 41; 39 *S. dauricus*, 2 *R. norvegicus*), Taigu County in Shanxi (n = 53, *R. norvegicus*), Nanning City in Guangxi (n = 74, *M. musculus*), Weihai City in Shandong (n = 227, *R. norvegicus*), Jiaxing City in Zhejiang (n = 119, *R. norvegicus*) and Xilingol League in Inner Mongolia (n = 22, *L. brandti*), China from June 2017 to November 2020. These rodents were captured by trapping method. The rodents had been euthanized by CO2 inhalation, and then the fresh feces sample (approximately 500 mg) was collected directly from the intestinal and rectal content of each rodent, and then was placed into ice boxes and sent to the laboratory. Information regarding sampling time, region, and species was recorded. This study was approved by the Ethics Committee of Qingdao Agricultural University.

### DNA Extraction and PCR Amplification

Genomic DNA was extracted from fecal sample of approximately 200 mg using the E.Z.N.A.^®^ Stool DNA Kit (Omega Biotek Inc., Norcross, GA, USA) according to the manufacturer’s instructions, and then was stored at -20°C prior to PCR. The prevalence and genotypes of *E. bieneusi* were identified by PCR amplification of the ITS gene according to the previous description (Zhao et al., 2018). *Cryptosporidium* spp. in the fecal samples was confirmed by PCR amplification of the SSU rRNA gene according to the previous report (Zhao et al., 2018). The positive and negative controls were included in each test. The secondary PCR products were observed using UV light after an electrophoretic analysis at a 1.5% agarose gel containing ethidium bromide.

### Sequence and Phylogenetic Analyses

The positive PCR specimens were sent to Sangon Biotech Company (Shanghai, China) for sequencing. A new PCR product should be sequenced if previously produced sequences had single nucleotide substitutions, insertions or deletions. The nucleotide sequences were aligned and analyzed with reference sequences by using the Clustal X 1.83 program and Basic Local Alignment Search Tool (BLAST) (https://blast.ncbi.nlm.nih.gov/), in order to determine the species/genotypes of *Cryptosporidium* spp. and *E. bieneusi*. The phylogenetic trees were reconstructed with Mega 5.0 using neighbor-joining (NJ) method under Kimura 2-parameter model (1,000 replicates). All nucleotide sequences were deposited in GenBank with accession numbers MT647749 – MT647806 and OK117929 – OK117932 for *E. bieneusi*, and MT561508 – MT561533 for *Cryptosporidium* spp.

### Statistical Analysis

The statistical analysis for the prevalence of *E*. *bieneusi* and *Cryptosporidium* in wild rodents from different region, season, sampling year, and species were performed by using χ2 test in SAS version 9.1 (SAS Institute, Cary, NC, USA). The results were considered to be statistically significant when *P* < 0.05. Odds ratios (ORs) and their 95% confidence intervals (95% CIs) were also calculated to compare the magnitude of various risk factors for *E*. *bieneusi* and *Cryptosporidium* prevalence.

## Results

### Prevalence of *Cryptosporidium *spp. and *E. bieneusi*

In the present study, 18 out of 536 (3.4%) fecal samples were identified as *Cryptosporidium*-positive (**Table 1**). The prevalence rates of *Cryptosporidium* in different species of rodents were 15% (6/401) in *R. norvegicus*, 9.5% (7/74) in *M. musculus*, 12.8% (5/39) in *S. dauricus*, and 0% (0/22) in *L. brandti* (**Table 1**). Moreover, the prevalence of *Cryptosporidium* in different regions ranged from 0% in Inner Mongolia (0/22) and Shandong (0/227) to 12.2% in Heilongjiang (5/41) (**Table 1**). Furthermore, the prevalence in different collection years ranged from 0% to 12.8% (**Table 1**). The prevalence of *Cryptosporidium* in rodent feces collected in autumn (3.7%, 12/321) was slightly higher than that in summer (2.8%, 6/215) (**Table 1**).

**Table 1:** Prevalence, associated factors, and distribution of *Cryptosporidium* spp. in rodents.

	No. positive/No. tested	Prevalence (%, 95% CI)	Species/Genotype	OR (95% CI)	*P*
**Area**					0.37
Zhejiang	4/119	3.4% (0.1-6.6)	*Cryptosporidium* sp. novel2 (n=4)	0.25 (0.06-10.98)	
Heilongjiang	5/41	12.2% (1.7-22.7)	*Cryptosporidium felis* (n=1), *Cryptosporidium* sp. novel1 (n=4)	Reference	
Shanxi	2/53	3.8% (0.0-9.1)	*Cryptosporidium* sp. novel2 (n=2)	0.28 (0.52-21.54)	
Guangxi	7/74	9.5% (2.6-16.3)	*Cryptosporidium viatorum* (n=1), *Cryptosporidium* sp. rat genotype II/III (n=5), *Cryptosporidium* sp. novel2 (n=1)	0.75 (0.22-2.54)	
Inner Mongolia	0/22	0	–	–	
Shandong	0/227	0	–	–	
**Rodent species**					0.001
*Rattus norvegicus*	6/401	1.5% (0.3-2.7)	*Cryptosporidium* sp. novel2 (n=6)	0.10 (0.0.-0.36)	
*Mus musculus*	7/74	9.5% (2.6-16.3)	*Cryptosporidium viatorum* (n=1), *Cryptosporidium* sp. novel2 (n=1), *Cryptosporidium* sp. rat genotype II/III (n=5)	0.71 (0.21-2.41)	
*Spermophilus dauricus*	5/39	12.8% (1.8-23.8)	*Cryptosporidium felis* (n=1), *Cryptosporidium* sp. novel1 (n=4)	Reference	
*Lasiopodomys brandti*	0/22	0		–	
**Sampling years**					< 0.01
2017	7/74	9.5% (2.6-16.3)	*Cryptosporidium viatorum* (n=1), *Cryptosporidium* sp. rat genotype II/III (n=5), *Cryptosporidium* sp. novel2 (n=1)	Reference	
2018	6/196	3.1% (0.6-5.5)	*Cryptosporidium* sp. novel2 (n=6)	0.30 (0.10-0.93)	
2019	5/39	12.8% (1.8-23.8)	*Cryptosporidium felis* (n=1), *Cryptosporidium* sp. novel1 (n=4)	1.41 (0.42-4.77)	
2020	0/227	0		–	
**Seasons**					0.77
Summer (6-8 months)	6/215	2.8% (0.6-5.0)	*Cryptosporidium* sp. novel2 (n=6)	Reference	
Autumn (9-11 months)	12/321	3.7% (1.7-5.8)	*Cryptosporidium viatorum* (n=1), *Cryptosporidium felis* (n=1), *Cryptosporidium* sp. rat genotype II/III (n=5), *Cryptosporidium* sp. novel1 (n=4), *Cryptosporidium* sp. novel2 (n=1)	1.16 (0.43-3.14)	
**Total**	18/536	3.4% (1.8-4.9)	*Cryptosporidium viatorum* (n=1), *Cryptosporidium felis* (n=1), *Cryptosporidium* sp. rat genotype II/III (n=5), *Cryptosporidium* sp. novel1 (n=4), *Cryptosporidium* sp. novel2 (n=7)		

Among 536 rodents, 62 samples (11.6%) were detected to be *E. bieneusi*-positive in three rodent species, with 13.3% (53/399) in *R. norvegicus*, 6.8% (5/74) in *M. musculus*, and 9.8% (4/41) in *S. dauricus* (**Table 2**). The highest prevalence of *E. bieneusi* was found in Shanxi (37.7%, 20/53), and followed by Zhejiang (24.4%, 29/119), Heilongjiang (9.8%, 4/41), Guangxi (6.8%, 5/74), and Shandong (1.4%, 4/227) (**Table 2**). The prevalence of *E. bieneusi* was 6.8% (5/74), 20.9%, (49/235) 9.8% (4/41), and 1.4% (4/227) in rodents collected in 2017, 2018, 2019, and 2020, respectively (**Table 2**). The prevalence of *E. bieneusi* in rodents was 22.8% in summer (49/215) and 4.0% in autumn (13/321), respectively (**Table 2**).

**Table 2:** Prevalence, associated factors, and distribution of *E. bieneusi* in rodents.

	No. positive/No. tested	Prevalence (%, 95% CI)	Genotype	OR (95% CI)	*P*
**Area**					0.00
Zhejiang	29/119	24.4% (16.5-32.2)	D (n=6), EbpA (n=3), EbpC (n=12), ZJR1 (n=1), ZJR2 (n=1), ZJR3 (n=1), ZJR4 (n=1), ZJR5 (n=1), ZJR6 (n=1), ZJR7 (n=2)	4.47 (1.64-12.08)	
Heilongjiang	4/41	9.8% (0.3-19.2)	EbpC (n=1), HLJC1 (n=2), HLJC2 (n=1)	1.49 (0.38-5.90)	
Shanxi	20/53	37.7% (24.2-51.2)	EbpA (n=3), EbpC (n=7), D (n=9), XJP-II (n=1)	8.36 (2.89-24.24)	
Guangxi	5/74	6.8% (0.9-12.6)	GXM1 (n=5)	Reference	
Inner Mongolia	0/22	0	–	–	
Shandong	4/227	1.4% (0.0-2.9)	NCF2 (n=1), SDR1(n=1), D (n=2)	0.25 (0.07-0.95)	
**Rodent species**					0.48
*Rattus norvegicus*	53/399	13.3% (9.9-16.6)	EbpA (n=6), EbpC (n=19), D (n=17), ZJR1 (n=1), ZJR2 (n=1), ZJR3 (n=1), ZJR4 (n=1), ZJR5 (n=1), ZJR6 (n=1), ZJR7 (n=2), XJP-II (n=1), NCF2 (n=1), SDR1(n=1)	1.33 (0.46-3.90)	
*Mus musculus*	5/74	6.8% (0.9-12.6)	GXR1 (n=5)	0.63 (0.16-2.51)	
*Spermophilus dauricus*	4/41	9.8% (0.3-19.2)	EbpC (n=1) HLJC1 (n=2) HLJC2 (n=1)	Reference	
*Lasiopodomys brandti*	0/22	0	–	–	
**Sampling years**					0.00
2017	5/74	6.8% (0.9-12.6)	GXM1 (n=5)	Reference	
2018	49/235	20.9% (15.6-26.1)	EbpA (n=6), EbpC (n=19), D (n=15), ZJR1 (n=1), ZJR2 (n=1), ZJR3 (n=1), ZJR4 (n=1), ZJR5 (n=1), ZJR6 (n=1), ZJR7 (n=2), XJP-II (n=1)	4.60 (1.76-12.06)	
2019	4/41	9.8% (0.3-19.2)	EbpC (n=1), HLJC1 (n=2), HLJC2 (n=1)	1.58 (0.40-6.25)	
2020	4/227	1.4% (0.0-2.9)	D (n=2), NCF2 (n=1), SDR1 (n=1)	0.25 (0.07-0.95)	
**Seasons**					0.00
Summer (6-8 months)	49/215	22.8% (17.1-28.4)	EbpA (n=6), EbpC (n=19), D (n=15), ZJR1 (n=1), ZJR2 (n=1), ZJR3 (n=1), ZJR4 (n=1), ZJR5 (n=1), ZJR6 (n=1), ZJR7 (n=2), XJP-II (n=1)	Reference	
Autumn (9-11 months)	13/321	4.0% (1.9-6.2)	GXM1 (n=5), EbpC (n=1), HLJC1 (n=2), HLJC2 (n=1), D (n=2), NCF2 (n=1), SDR1(n=1)	0.12 (0.06-0.23)	
**Total**	62/536	11.6% (8.9-14.3)	EbpA (n=6), EbpC (n=20), D (n=17), XJP-II (n=1), NCF2 (n=1), ZJR1 (n=1), ZJR2 (n=1), ZJR3 (n=1), ZJR4 (n=1), ZJR5 (n=1), ZJR6 (n=1), ZJR7 (n=2), GXM1 (n=5), HLJC1 (n=2), HLJC2 (n=1), SDR1(n=1)		

*E. bieneusi* and *Cryptosporidium* spp. coinfection was found in three wild rodents in this study. All of them were *R. norvegicus* collected in 2018. Two were collected from Zhejiang Province, and the remaining one was collected from Shanxi Province.

### Distribution of *Cryptosporidium* spp. and *E. bieneusi*

**Figure 1:**
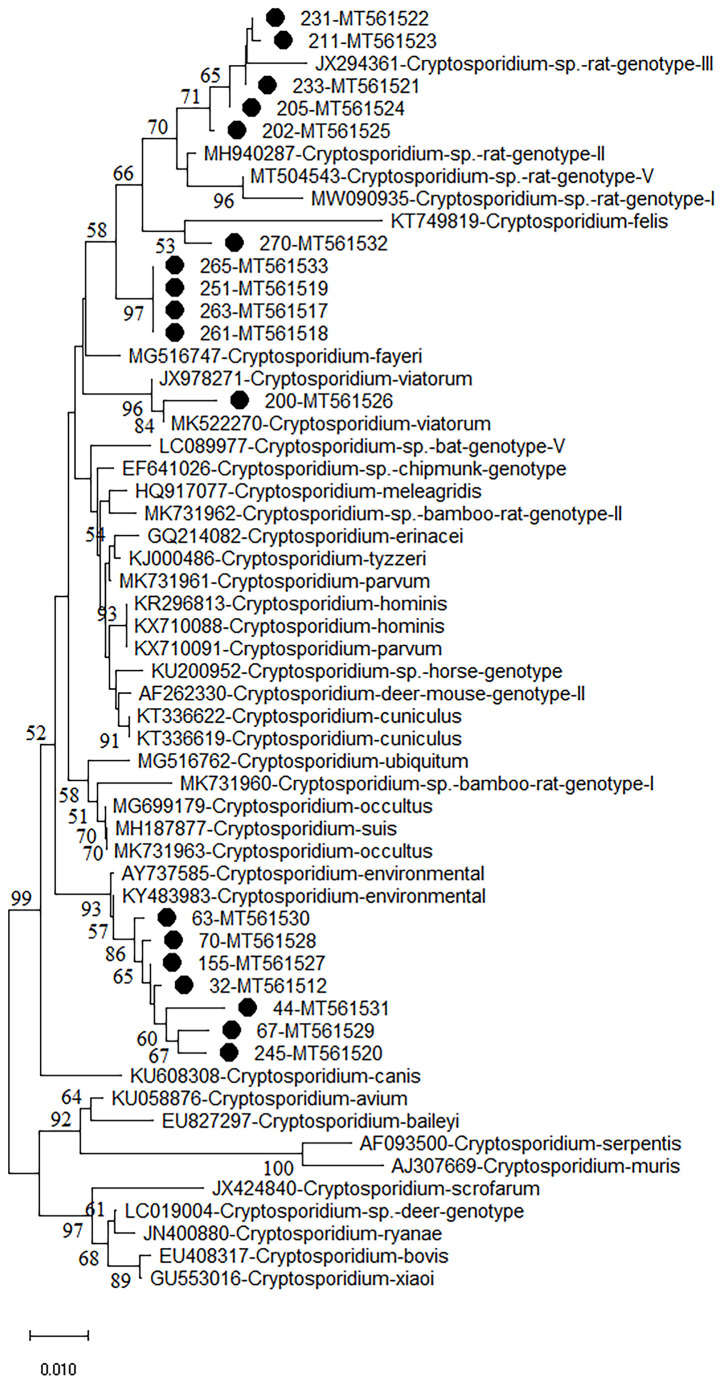
Phylogenetic analyses of SSU rRNA gene of *Cryptosporidium* spp. using neighbor-joining (NJ) method (Kimura 2-parameter model, 1,000 replicates). Bootstrap values below 50% are not shown. *Cryptosporidium* isolates identified in the present study are pointed out by solid circles.

*Cryptosporidium* sp. rat genotype II/III, *Cryptosporidium felis*, and *Cryptosporidium viatorum* were identified in the investigated rodents through the analysis of SSU rRNA gene of *Cryptosporidium*. Furthermore, two *Cryptosporidium* genotypes with uncertain species status were observed (**Figure 1** and **Table 1**). *Cryptosporidium* sp. novel1 and *C. felis* were found in *S. dauricus* in Heilongjiang. *C. viatorum* and *Cryptosporidium* sp. rat genotype II/III were only identified in *M. musculus* in Guangxi. *Cryptosporidium* sp. novel2 was found in three provinces Zhejiang (*R. norvegicus*), Shanxi (*R. norvegicus*), and Guangxi (*M. musculus*) (**Table 1**).

**Figure 2:**
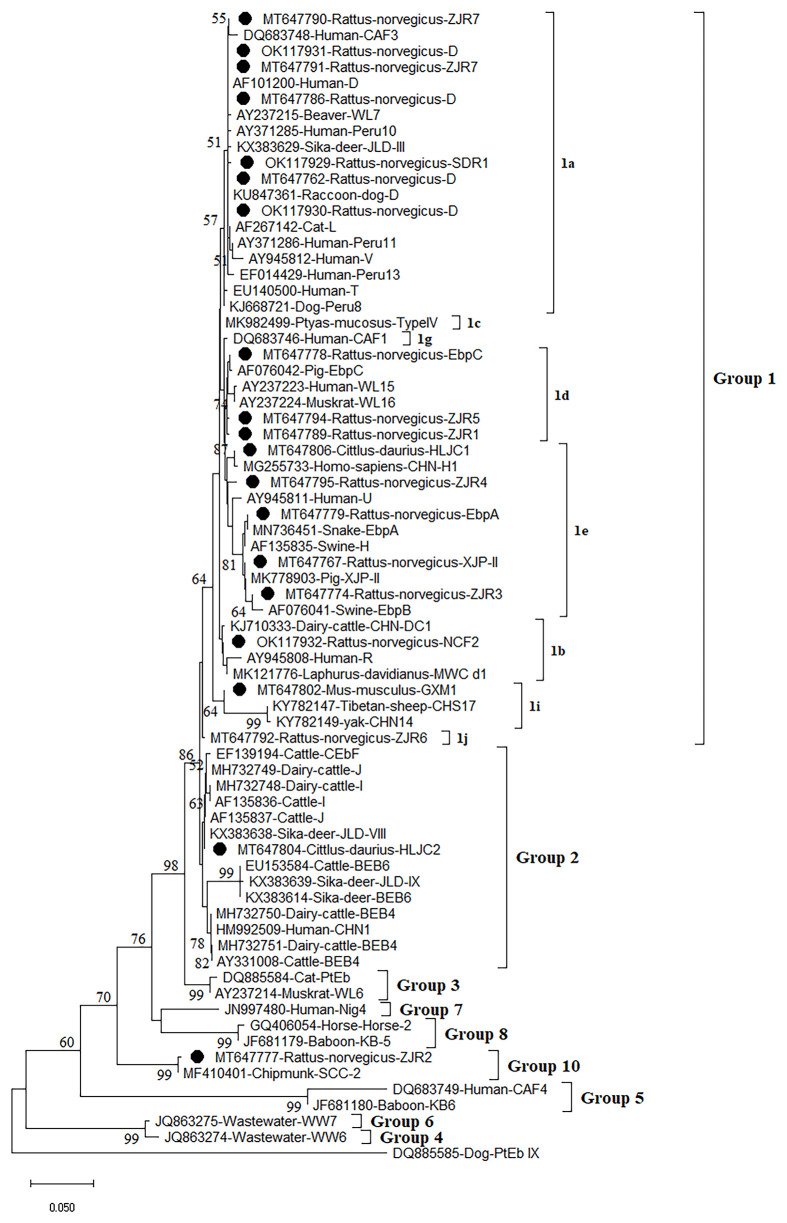
Phylogenetic analyses of ITS gene of *Enterocytozoon bieneusi* using neighbor-joining (NJ) method (Kimura 2-parameter model, 1,000 replicates). Bootstrap values below 50% are not shown. *E. bieneusi* isolates identified in the present study are pointed out by solid circles.

A total of 16 *E. bieneusi* genotypes were identified in this study, including 5 known genotypes (XJP-II, EbpC, EbpA, D, and NCF7) and 11 novel genotypes (ZIR1 to ZJR7, GXM1, HLJC1, HLJC2, and SDR1) (**Figure 2** and **Table 2**). Among them, genotype D was found in *R. norvegicus* in Zhejiang, Shanxi, and Shandong. EbpA was only found in *R. norvegicus* in Zhejiang and Shanxi, whereas EbpC was identified in Zhejiang (*R. norvegicus*), Shanxi (*R. norvegicus*), and Heilongjiang (*S. dauricus*). Moreover, NCF2 (*R. norvegicus* in Shandong), XJP-II (*R. norvegicus* in Shanxi), ZIR1 to ZJR7 (*R. norvegicus* in Zhejiang), GXM1 (*M. musculus* in Guangxi), HLJC1 (*S. dauricus* in Heilongjiang), HLJC2 (*S. dauricus* in Heilongjiang), and SDR1 (*R. norvegicus* in Shandong) were only found in one province (**Table 2**).

### Phylogenetic Relationships of *Cryptosporidium* spp. and *E. bieneusi*

The phylogenetic analysis of various *Cryptosporidium* species/genotypes showed two uncertain species status and three known species/genotypes (**Figure 1**). The sequences of *Cryptosporidium* sp. novel2, including seven *Cryptosporidium* spp. sequences (isolates 32, 44, 63, 67, 70, 155, and 245), were clustered with *Cryptosporidium* spp. sequences identified from environmental samples (**Figure 1**). Five sequences (isolates 202, 205, 211, 231, and 233) were clustered with *Cryptosporidium* sp. rat genotype II/III in a same clade (**Figure 1**). Sequences of isolates 251, 261, 263, and 265 (*Cryptosporidium* sp. novel1) were grouped into a novel separate clade (**Figure 1**). Sequences of isolates 270 and 200 were clustered with that of *C. felis* and *C. viatorum* in a same clade, respectively (**Figure 1**).

The Neighbor-Joining analysis for sequences of *E. bieneusi* species/genotypes obtained in this study revealed that 5 known genotypes and 11 novel genotypes (**Figure 2**). Fourteen genotypes (5 known genotypes and 9 novel genotypes) were divided into Group 1, with ZJR7, SDR1, and D in 1a, EbpC, ZJR5, and ZJR1 in 1d, HLJC1, ZJR4, EbpA, XJP-II, and ZJR3 in 1e, NCF2 in 1b, GXM1 in 1i, and ZJR6 in 1j (**Figure 2**). Furthermore, HLJC2 was grouped in Group 2, and ZJR2 was classified into Group 10 (**Figure 2**).

## Discussion

In this study, the total prevalence of *Cryptosporidium* spp. was 3.4% (18/536) in four rodent species (*R. norvegicus*, *M. musculus*, *L. brandti*, and *S. dauricus*), which was consistent with previous reports showing the prevalence rates ranged from 0.8% to 80.0% in a variety of rat species (Feng, 2010; Mirzaghavami et al., 2016; Wei et al., 2019), e.g., 1.5-38.0% in brown rats, 8.0-31.4% in mice, and 0.8-73.0% in voles (Feng, 2010; Wei et al., 2019; Ježková et al., 2021). The present study found that the prevalence rates of *Cryptosporidium* spp. in *R. norvegicus*, *M. musculus*, *L. brandti*, and *S. dauricus* were 1.5% (6/401), 9.5% (7/74), 0% (0/22), and 12.8% (5/39), respectively with statistical significance (*P* < 0.05). There was a 0.10- (OR = 0.10, 95% CI 0.0-0.36) and 0.71- (OR = 0.71, 95% CI 0.21-2.41) fold increase of *Cryptosporidium* spp. infection risk in *R. norvegicus* (1.5%, 95% CI 0.3-2.7), *M. musculus* (9.5%, 95% CI 2.6-16.3) compared with that in *S. dauricus* (12.8%, 95% CI 1.8-23.8). Furthermore, the prevalence of *E. bieneusi* in rodents varied in different countries, e.g., 87.5% in Peru (Cama et al., 2007), 28.6-42.9% in Poland (Perec-Matysiak et al., 2015), 1.1% in Slovakia (Danišová et al., 2015), 20.0-100% in USA (Roellig et al., 2015). In the present study, the overall *E. bieneusi* prevalence was 11.6% (62/536), with 13.3% (53/399) in *R. norvegicus*, 6.8% (5/74) in *M. musculus*, 9.8% (4/41) in *S. dauricus*, and 0% (0/22) in *L. brandti*. In China, *E. bieneusi* infection has also been reported in many rodent species, such as Bamboo rat (5.1%, 22/435; 15.4%, 18/117) (Wang et al., 2019; Zhao et al., 2020), Brown rat (7.9%, 19/242; 14.3%, 8/58) (Zhao et al., 2018; Zhao et al., 2020), Chinchilla (3.6%, 5/140) (Qi et al., 2015), Indo-Chinese forest rat (9.3%, 5/54) (Zhao et al., 2020), Asiatic brush-tailed porcupine (7.5%, 7/93) (Zhao et al., 2020), Bower’s white-toothed rat (31.6%, 37/117) (Zhao et al., 2020), Edward's long-tailed rat (7.9%, 3/38) (Zhao et al., 2020), Chipmunk (17.6%, 49/279 (Deng et al., 2018), Asian house rat (23.1%, 31/134) (Zhao et al., 2020), Chinese white-bellied rat (18.2% 6/33) (Zhao et al., 2020), Lesser rice-feld rat (36.4%, 16/44) (Zhao et al., 2020). Coinfection (n = 3) of *E. bieneusi* and *Cryptosporidium* spp. was also found in the present study. Different susceptibility of different rodent species, different sampling time and sample size, animal age, and animal welfare could affect the prevalence of *Cryptosporidium* spp. and *E. bieneusi* in different rodent species in different regions.

Although *Cryptosporidium* spp. in rodent feces collected in summer (6/215, 2.8%, 95% CI 0.6-5.0) has a slightly lower prevalence than those collected in autumn (12/321, 3.7%, 95% CI 1.7-5.8), the difference was not significant statistically (*P* = 0.77) (**Table 1**). Moreover, the temperature and humidity in summer (49/215, 22.8%, 95% CI 17.1-28.4) may be more suitable for the survival of *E. bieneusi* oocysts than in autumn (13/321, 4.0%, 95% CI 1.9-6.2), the infection risk of *E. bieneusi* had 0.12-fold increase (OR = 0.12, 95% CI 0.06-0.23) in rodent feces collected in autumn (4.0%, 95% CI 1.9-6.2) than that in summer (22.8%, 95% CI 17.1-28.4) in the investigated rodents (**Table 2**). The investigated rodents were more active in the summer temperature, which might be the other reason for these rodents to be infection and transmission increase. Other ecological factors such as climate, food resources, breeding, physical activity, etc, which might affect the accuracy of prevalence of the two pathogens, should also be investigated in the further study.

More than 30 *Cryptosporidium* species/genotypes have been identified in rodents. However, only five species/genotypes were identified in this study, including *C. viatorum, C. felis, Cryptosporidium* sp. rat genotype II/III*, Cryptosporidium* sp. novel1, and *Cryptosporidium* sp. novel2. Among them, *Cryptosporidium* sp. rat genotype II/III, previously reported in rodents (García-Livia et al., 2020; Ježková et al., 2021), was also identified in this study, which was further confirmed that *Cryptosporidium* sp. rat genotype II/III was one of the prevalent *Cryptosporidium* genotypes in rodents. Moreover, two uncertain species of *Cryptosporidium* (*Cryptosporidium* sp. novel1 and novel2) were also identified in this study. *Cryptosporidium* sp. novel1 (isolates 251, 261, 263, and 265) was grouped into a new separate clade. *Cryptosporidium* sp. novel2 (isolates 32, 44, 63, 67, 70, 155, and 245), grouped with *Cryptosporidium* environmental. The results indicate two new genotypes/species that have clustered a branch in phylogenetic analysis with environmental isolates of *Cryptosporidium* spp. One of the reasons that in environmental samples, it is difficult to determine the species and genotype is the simultaneous contamination of several species and genotypes in samples that after sequencing cannot detect a known species or genotype. Unfortunately, other genes such as COWP and HSP70 of the uncertain species have also not been successfully amplified. Thus, the investigation should be continue performed to further confirm whether presence of the two uncertain species of *Cryptosporidium* in wild rodents. *C. viatorum*, has been identified in humans (Insulander et al., 2013; Lebbad et al., 2013; Adamu et al., 2014; Ayinmode et al., 2014; De Lucio et al., 2016; Sanchez et al., 2017; Ukwah et al., 2017; Sannella et al., 2019)*. C. viatorum* was first found in travellers who returned to the United Kingdom from the Indian subcontinent, with clinical signs of diarrhea, fever, headache, abdominal pain, nausea, vomiting, and marked weight loss (Elwin et al., 2012). So far, *C. viatorum* has been documented in the following countries: Bangladesh, Ethiopia, Barbados, Kenya, Colombia, Nigeria, Pakistan, Guatemala, India, and Nepal (Insulander et al., 2013; Lebbad et al., 2013; Adamu et al., 2014; Ayinmode et al., 2014; De Lucio et al., 2016; Sanchez et al., 2017; Ukwah et al., 2017; Sannella et al., 2019). Besides, *C. viatorum* was also found in China, such as Hainan Province (*Leopoldamys edwardsi*), Guangdong Province (*Berylms bowersi*), and Chongqin*g* City (*Leopoldamys edwardsi*) in China, and in Australia (*Rattus lutreolus*) (Koehler et al., 2018; Chen et al., 2019; Zhao et al., 2019). C. felis has been widely reported in cats (Jiang et al., 2020), in addition to patients with HIV/AIDS in Peru, Ethiopia, Nigeria, Jamaica, and Portugal (Cama et al., 2003; Jiang et al., 2020). In this study, *C. viatorum* and *C. felis* were found in *M. musculus* and *S. dauricus*, which was worth for further research, e.g., whether wild rodents are potentially important reservoirs for *C. viatorum* and *C. felis* transmission to humans. More importantly, this is the first study showing existence of *Cryptosporidium* spp. in *S. dauricus*, which has expanded the host ranges of *Cryptosporidium*.

At present, more than 400 genotypes of *E. bieneusi* have been identified, most of which exhibit host specificity (Santín and Fayer, 2011; Wang S. N. et al., 2020). At least 48 genotypes of *E. bieneusi* infect both human and animals, bringing zoonoses risks (Li and Xiao, 2019). Through phylogenetic analysis, these genotypes were divided into at least 11 groups, e.g., Group 1 to Group 11 (Zhao et al., 2018; Wang S. N. et al., 2020). To date, some genotypes were found in rodents, of which 15 genotypes (CZ3, Peru6, BEB6, C, D, EbpA, EbpC, H, Peru8, Peru11, Peru16, PigITS5, S6, IV, and Nig7) were reported to infect human. In China, EbpA, EbpC, CHY1, N, D, Peru11, S7, SCC-2, PGP, Peru6, J, PigEBITS7, BR1 and BR2, Type IV, Peru8, ESH02, CHG5, HNR-I to HNR-VII, K, CQR-1, CQR-2, CQR-3, GDR-1, GDR-2, GDR-3. SCC-1, SCC-3, SCC-4, CHY1, Nig7 CHG9, ChG14, BEB6, CHG2, SC02, CE01 and CE02 genotypes were reported in rodents (Feng et al., 2009; Zhao et al., 2018; Wang et al., 2019; Li J. et al., 2020; Wang J. et al., 2020; Zhao et al., 2020). However, only 5 known genotypes (XJP-II, EbpC, EbpA, D, and NCF7) and 11 novel genotypes (ZIR1 to ZJR7, GXM1, HLJC1, HLJC2, and SDR1) were identified in the present study. Among them, 14 genotypes were clustered into a highly-supported monophyletic clade (Group 1), indicating that these genotypes are human-pathogenic types and may cause infection between humans and rodents, thus becoming a public health significance. This was the first record of *E. bieneusi* in *S. dauricus*. Eleven novel genotypes (ZIR1 to ZJR7, GXM1, HLJC1, HLJC2, and SDR1) were recorded in rodents for the first time. Of which, ZJR1, ZJR3, ZJR4, ZJR5, ZJR6, ZJR7, SDR1, HLJC1, and GXM1 were grouped into Group 1 (**Figure 2**), thus suggesting that rodents (*R. norvegicus*, *M. musculus*, and *S. dauricus*) may play an important role in the transmission of *E. bieneusi* between rodents and humans. Genotype XJP-II was previously found in pigs in Xinjiang (Li D. F. et al., 2019b), and NCF2 was also identified in farmed foxes (*Vulpes lagopus*) (Zhang et al., 2016; Ma et al., 2020) and raccoon dogs (*Nyctereutes procyonoides*) (Xu et al., 2016) in China, Kangaroo in Australia (Zhang Y. et al., 2018). Genotypes EbpC, EbpA, and D were frequently found in humans and a broad range of animals (Wang et al., 2013; Liu et al., 2017; Qi et al., 2018; Zhang X. X. et al., 2018; Zou et al., 2018; Wang H. et al., 2020; Wang Y. et al., 2020; Yu et al., 2020). The results showed that natural transmission of *E. bieneusi* among rodents, humans and many other animals may occur. More importantly, the three ITS genotypes were also found in water in China, which should be paid more attention to prevent the water-borne transmission of *E. bieneusi* (Hu et al., 2014).

Collectively, the present study firstly demonstrated that existence of *Cryptosporidium* spp. (3.4%, 18/536) and *E. bieneusi* (11.6%, 62/536) in rodents in Shanxi, Guangxi, and Zhejiang, China. Three known *Cryptosporidium* species/genotypes (*C. viatorum*, *C. felis*, and *Cryptosporidium* sp. rat genotype II/III), two uncertain *Cryptosporidium* species/genotypes (*Cryptosporidium* sp. novel1 and *Cryptosporidium* sp. novel2), 5 known *E. bieneusi* genotypes (XJP-II, EbpC, EbpA, D, and NCF7) and 11 novel *E. bieneusi* genotypes (ZJR1 to ZJR7, GXM1, HLJC1, HLJC2, and SDR1) were identified in the investigated rodents, suggesting rodents can act as a potential source of human and animal infections. *E. bieneusi* was more prevalent in *R. norvegicus*, whereas *Cryptosporidium* spp. was more frequently identified in *S. dauricus*. The present study also demonstrated that *S. dauricus* was the host of *E. bieneusi* and *Cryptosporidium* spp. for the first time. This study expanded the host range of these two parasites, which not only provided basic data for distribution of *E. bieneusi* and *Cryptosporidium* genotypes/species, but also provided foundation data for the prevention and control of *E. bieneusi* and *Cryptosporidium* spp. in China.

## Data Availability Statement

The datasets presented in this study can be found in online repositories. The names of the repository/repositories and accession number(s) can be found below: https://www.ncbi.nlm.nih.gov/genbank/, MT647749-MT647806, OK117929-OK117932, MT561508-MT561533.

## Ethics Statement

This study was approved by the Ethics Committee of Qingdao Agricultural University.

## Author Contributions

QZ, Y-CW, and H-TS conceived and designed the study and critically revised the manuscript. H-BN, S-YQ, DY, Z-HF, Z-HG, H-XW, H-YQ, and NX collected the samples. Z-YS, MZ, and Y-ZS performed the experiments. H-BN, Y-ZS, and S-YQ analyzed the data and drafted the manuscript. All authors contributed to the article and approved the submitted version.

## Funding

This work was supported by the National Key R&D Program of China, the National Innovation and Entrepreneurship Training Program for College Students of Shandong Province (202110435018), the State Key Laboratory of Veterinary Etiological Biology, Lanzhou Veterinary Research Institute, Chinese Academy of Agricultural Sciences (SKLVEB2019KFKT012), the Wild Animal Disease Monitoring and Early Warning System Maintenance Project (2130211), and the Research Foundation for Distinguished Scholars of Qingdao Agricultural University (665-1120046).

## Conflict of Interest

Author Y-CW is employed by Veterinary Department, Muyuan Foods Co., Ltd.

The remaining authors declare that the research was conducted in the absence of any commercial or financial relationships that could be construed as a potential conflict of interest.

## Publisher’s Note

All claims expressed in this article are solely those of the authors and do not necessarily represent those of their affiliated organizations, or those of the publisher, the editors and the reviewers. Any product that may be evaluated in this article, or claim that may be made by its manufacturer, is not guaranteed or endorsed by the publisher.
